# Microencapsulation of *Tecoma stans* Extracts: Bioactive Properties Preservation and Physical Characterization Analysis

**DOI:** 10.3390/foods13071001

**Published:** 2024-03-25

**Authors:** Jair R. García-Jiménez, María L. Luna-Guevara, Juan J. Luna-Guevara, Lilia A. Conde-Hernández, María E. Ramos-Cassellis, Heriberto Hernández-Cocoletzi

**Affiliations:** 1 Faculty of Chemical Engineering, Meritorious Autonomous University of Puebla, Av San Claudio and 18 Sur, Ciudad Universitaria, Puebla 72570, Mexico; jair.garciaj@alumno.buap.mx (J.R.G.-J.); lilia.conde@correo.buap.mx (L.A.C.-H.); elena.ramos@correo.buap.mx (M.E.R.-C.); heriberto.hernandez@correo.buap.mx (H.H.-C.); 2 Posgraduate in Science in Sustainable Agroecosystem Management, Edificio VAL 1, Ecocampus Valsequillo, San Pedro Zacachimalpa, Puebla 72960, Mexico

**Keywords:** *Tecoma stans*, spray drying, microencapsulation, antioxidant activity, hypoglycemic activity, bioactive compounds, medicinal plants

## Abstract

Bioactive compounds from medicinal plants have applications in the development of functional foods. However, since they are unstable, encapsulation is used as a conservation alternative. This work aimed to assess the bioactive properties (antioxidant and hypoglycemic) of different extracts, including the infusion, as well as their spray-dried microencapsulates from *Tecoma stans* leaves. A factorial design was proposed to determine the best extraction conditions, based on ABTS and DPPH inhibition. Maltodextrin (MD), arabic gum (AG), and a 1:1 blend (MD:AG) were used as encapsulating agents. Moreover, characterization through physicochemical properties, gas chromatography/mass spectrometry (GC-MS) and scanning electron microscopy (SEM) of the best two powders based on the bioactive properties were analyzed. The results showed that the combination of stirring, water, and 5 min provided the highest inhibition to ABTS and DPPH (35.64 ± 1.25 mg Trolox/g d.s. and 2.77 ± 0.01 g Trolox/g d.s., respectively). Spray drying decreased the antioxidant activity of the extract while preserving it in the infusion. The encapsulated infusion with MD:AG had the highest hypoglycemic activity as it presented the lowest glycemic index (GI = 47). According to the results, the microencapsulates could potentially be added in foods to enhance nutritional quality and prevent/treat ailments.

## 1. Introduction

*Tecoma stans*, a species native to México and Central America, is a medicinal plant widely used to treat several conditions. In México, it is mainly used to treat diabetes through leaf infusion intake [1,2]. As a result of its broad usage in traditional medicine, the bark, flower, and leaves of the plant have been subject to in vivo and in vitro analysis, showing pharmacological properties that include antidiabetic and hypoglycemic [3,4,5], antioxidant [6,7,8], antimicrobial and antifungal [9,10,11,12], anti-inflammatory [13,14], cardioprotective [15], hepatoprotective [16], antiulcer [17], and anticancer [18].

Historically, plants have been employed since ancient times [19]. Currently, roughly 60% of the population of the world and 80% of the population in underdeveloped countries use medicinal plants to alleviate diseases, according to data from the WHO [20]. The therapeutic effect of plants is due to the presence of bioactive compounds. These compounds have raised interest in being investigated by the scientific community as well as by the food and pharmaceutical industries, as they can be natural and safer alternatives to prevent and treat chronic diseases. In the food industry, compounds with biological activity from plants could be potentially used as preservatives and functional ingredients. However, high dose requirements in food matrices, low bioavailability, the unknown mode of action, and the availability of raw material are some hurdles to their utilization in the development of functional foods [21]. Likewise, many bioactives are sensitive to conditions that cause their degradation, like pH, temperature, light, and moisture [22].

The extraction and separation process of bioactives from plant tissue through appropriate conditions and solvents is considered the most critical stage in the analysis of compounds exhibiting biological activity since it influences the quality and quantity of the extracted bioactives and the next steps of isolation and characterization. That is why the suitable solvent and method election play a crucial role in the efficient extraction of bioactive compounds from natural sources [23,24,25].

Encapsulation is the process where liquid, solid, and gas substances are covered within a food-grade material layer, forming a wall. Such substances can be heat-sensitive, like bioactive compounds. The resulting capsules can be produced on nano (<1 μm) or micro (1–1000 μm) scales. This process can confer protection by improving solubility, stability, and bioavailability, as well as controlling the release of bioactive compounds and masking undesired odors and off-flavors [26,27]. Moreover, encapsulated components can be eventually incorporated into food systems [28]. The factors to consider when selecting the appropriate encapsulation method include the type of substance (core), the carrier agent, and the intended application. Nevertheless, spray drying is the established method in the food industry, as around 90% of microcapsules are processed through this technology [29,30]. Uncomplicated storage, high process yield, flexibility, low energy consumption, low water activity, and short processing time are the advantages of the powders obtained via spray drying [31].

Several materials are used to encapsulate bioactive compounds. The coating material directly affects the encapsulation efficiency and stability. Therefore, the type, origin, and properties of the natural bioactive ingredients and the food system properties into which the microcapsules are intended to be added must be considered when selecting a suitable wall material. Wall materials are generally composed of carbohydrates, proteins, and lipids. Polymers such as maltodextrin, arabic gum, modified starches, chitosan, and mixtures are commonly used as coating materials [26,32].

This work aimed to encapsulate *Tecoma stans* leaf extracts by spray drying using maltodextrin, arabic gum, and a combination of both agents. In addition, the antioxidant and hypoglycemic activities, as well as the physical and compound characterization of the powders were analyzed. As of now, there is no research on the encapsulation of *Tecoma stans* leaf extracts.

## 2. Materials and Methods

### 2.1. Plant Material

The leaves were collected from an identified *Tecoma stans* (L.) Juss. ex kunth var. stans (*Bignoniaceae*) tree at the Botanical Garden of the Meritorious Autonomous University of Puebla (Puebla, México).

### 2.2. Sample Preparation

The leaves free of physical damage were selected. Fresh leaves were used in the infusion while grounded dried leaves (30 °C) were used for the other extracts. The grounded sample was passed through an 80-mesh sieve (177 μm). The pulverized sample was kept in amber bottles protected from light and stored at room temperature.

### 2.3. Extraction

#### 2.3.1. Factorial Design

50 mg of the dried grounded sample was used for the extraction based on a 2^3^ factorial design model, in which two methods (stirring and ultrasound bath performed with a working frequency of 40 kHz and an ultrasonic power of 180 W), 10 mL of two solvents (distilled water and ethanol) and two different times (5 and 15 min) were considered (Table 1).

#### 2.3.2. Infusion Preparation

The infusion was prepared as it is generally conducted in Mexican traditional medicine. Moreover, 35 g of fresh leaves were boiled with 1 L of distilled water for 4 min. The infusion was left to stand for 1 h and then filtered. The infusion was protected from light and stored at 4 °C until use.

### 2.4. Antioxidant Activity

The antioxidant activity was determined via the ABTS (2,2′-azino-di-(3-ethylbenzthiazoline sulfonic acid)) (Sigma-Aldrich, St. Louis, MO, USA) and DPPH (2,2-difenil-1-pricrilhidrazil)) (Sigma-Aldrich, St. Louis, MO, USA) methods.

#### 2.4.1. ABTS

The ABTS method was performed according to the methodology of Conde-Hernández and Guerrero-Beltrán [33]. The radical was formed by letting stand, at room temperature and protected from light for 16 h, a mixture of 3.3 mg of potassium persulfate and 19.4 mg of the ABTS reactive, dissolved in 5 Ml of distilled water. The mixture was then diluted in ethanol until obtaining an initial absorbance (A_i_) of 0.7 ± 0.02 analyzed at 754 nm with a UV-Vis spectrophotometer (Jenway, 6405 UV/Vis, Princeton, NJ, USA). For the final absorbance (A_f_), 80 μL of the extract was mixed with 3920 μL of the solution radical ABTS-ethanol and measured at 754 nm. Both A_i_ and A_f_ measurements were made after 7 min of reaction. The ABTS inhibition percentage was calculated with Equation (1).

ABTS Inhibition %= ((A_i_ − A_f_)/A_i_) × 100 (1)

#### 2.4.2. DPPH

The DPPH method was performed according to the methodology of Bajalan et al. [34], with some modifications, in which a methanolic DPPH solution (6.08 × 10^−8^ mM) was used. The control solution (A_b_) was a mixture of 3900 μL of the methanolic DPPH solution and 100 μL of methanol. The final absorbance (A_f_) solution was obtained by mixing 3900 μL of the methanolic DPPH solution with 100 μL of the extract. Both A_b_ and A_f_ measurements were analyzed at 517 nm with a UV-Vis spectrophotometer (Jenway, 6405 UV/Vis, Princeton, NJ, USA) after 30 min of reaction. The DPPH inhibition percentage was calculated with Equation (2).

DPPH Inhibition %= (1 − (A_f_/A_b_)) × 100 (2)

#### 2.4.3. Standard Curve

A Trolox (6-hydroxy-2,5,7,8 tetramethylchromane-2-carboxylic acid) curve was used in both methods to report the antioxidant activity as mg Trolox/g of dry solids (mg Trolox/g d.s.). The concentration range used was 0–0.2 mg/mL (R^2^ = 0.99) and 0–14 mg/L (R^2^ = 0.99) for the ABTS and DPPH radicals, respectively.

### 2.5. Hypoglycemic Activity

The hypoglycemic activity of the samples was analyzed through the measurement of glucose release reported byJenkins [35]. Initially, a 12 kDa retention membrane (Sigma-Aldrich, St. Louis, MO, USA) of around 12 cm in length was hydrated with distilled water. Once hydrated, 200 mg (d.b.) of sample diluted in 20 mL of a glucose solution (40 mmol/L) was left to settle for 30 min and then placed into the membrane. The membrane was dipped into an Erlenmeyer flask containing 100 mL of distilled water and incubated (37 °C; 120 rpm) for 120 min. During this time, aliquots were taken every 15 min.

Aliquots were analyzed with glucose oxidase and a standard through the GLUCOSE-LQ kit (SPINREACT, Sant Esteve de Bas, Spain), measuring the absorbances at 500 nm with a UV-Vis spectrophotometer (Jenway, 6405 UV/Vis, Long Branch, NJ, USA). The absorbances were used to calculate the area under the curve (∆) with the trapezoidal rule. The amount of released glucose was estimated with Equation (3). The same procedure was followed for white bread, which was used as a reference sample.

Released glucose = (∆ sample/∆ standard) × n,(3)

where n is the standard concentration (n = 100 mg/dL; 5.56 mmol/L).

Additionally, from the released glucose, the glycemic index (GI) was estimated with Equation (4), as described in ISO standard 266642:2010 [36].

GI = (∆ sample (mmol/L)/∆ white bread (mmol/L)) × 100(4)

### 2.6. Spray Drying Microencapsulation

#### 2.6.1. Mixture Preparation

The best extraction treatment selected from the factorial design model and the infusion were microencapsulated. Maltodextrin (MD) (dextrose equivalent 4.0–7.0) (Sigma-Aldrich, USA), arabic gum (AG) (Meyer, Mexico city, México), and a maltodextrin–arabic gum 1:1 blend (MD:AG) were used as the encapsulating agents. The feed solution between the extract/infusion (core) and the encapsulating agents consisted of 8% agents and 92% core.

The mixtures were homogenized at room temperature using a magnetic stirrer reaching 700 rpm/min for approximately 45 min until complete dissolution.

#### 2.6.2. Spray Drying Process

The mixtures were spray-dried using a SEV Prendo spray dryer (Puebla, México) at a feed flow rate of 3 ± 1 g/min, which was provided by a peristaltic pump. Temperatures at the entrance and exit of the system were 160 °C and 96 °C, respectively.

### 2.7. Physicochemical Characterization

The yield, moisture, water activity, wettability, apparent and bulk density, and color of the microcapsules that showed the best bioactive properties were determined, as described next.

#### 2.7.1. Yield

The yield was calculated with Equation (5), proposed by Fang and Bhandari [37], under some modifications.

Yield (%) = (powder obtained by spray drying (g)/(extract (g) + encapsulating agent (g))) × 100(5)

#### 2.7.2. Moisture

The moisture content was obtained gravimetrically by drying 1 g of powder obtained in a convection oven at 105 °C until reaching a constant weight [38].

#### 2.7.3. Water Activity (Aw)

The water activity of the powders was measured with a hygrometer (Aqualab Series 3TE, Washington, DC, USA). 

#### 2.7.4. Wettability

The wettability was determined following the methodology described by Visotto et al. [39], measuring the time when 0.1 g of powder spread on the surface of 100 mL of distilled water at 25 °C, fully submerged in the liquid.

#### 2.7.5. Bulk and Tapped Density

The bulk density (Equation (6)), in which the volume (V0) of a certain amount of mass (m0) of powder is measured, was calculated according to the method by Tonon et al. [40]. For the tapped density (Equation (7)), the methodology reported by Jangam and Thorat [41] was followed, tapping the same powder until it reached a compacted volume (Vc).

Bulk density = (m0/V0) × 100(6)

Tapped density = (m0/Vc) × 100(7)

#### 2.7.6. Color

The color of the microencapsulates was analyzed with a colorimeter (HunterLab, Color Flex 45/0 Spectrophotometer, Washington, DC, USA), considering the values of the chromatids L* (light/dark), a* (red/green), and b*(yellow/blue).

### 2.8. Compound Identification

Likewise, the powders with the best bioactive properties were analyzed through gas chromatography/mass spectrometry (GC-MS) to characterize their volatile compounds. Ethanol, hexane, and ethyl acetate were used as solvents, using a gas chromatograph equipped with a 5975 quadrupole mass selective detector (Agilent Technologies 6850N GC, Santa Clara, CA, USA) along with an HP5-MS column (30 m in length and 0.25 mm in diameter). The set conditions were as follows: helium as the carrier gas at a flow rate of 15.5 mL/min, an injector temperature of 250 °C, an injection volume of 1 μL, a split ratio of 10:1, and the programmed temperature starting at 60 °C and increasing at 4 °C/min until it reached 250 °C. The ionization energy was 43–350 *m*/*z*. Volatile compound identification was carried out by comparing their mass spectra with information published in the literature.

### 2.9. Micrographs

A scanning electron microscope (SEM) (JEOL JSM-6610-LV, Akyshama, Japan) was employed to assess the morphology and size of the microcapsules, using the backscattered electron technique with a 10 kV acceleration. The images were processed using the ImageJ software version 1.54g (National Institutes of Health).

### 2.10. Data Analysis

The experiments were performed in triplicate and presented as mean ± S.D. The data were analyzed with the statistical software Minitab 18, using an analysis of variance (ANOVA) (α = 0.05) and the Tukey test to determine significant differences (*p* < 0.05). Regarding the factorial design, the main and interaction effects plots and the Pareto chart of the significance rank of the main and interaction effects of extraction factors on antioxidant activity were analyzed.

## 3. Results and Discussion

### 3.1. Antioxidant Activity of Extracts Based on Factorial Design

According to Vega et al. [42], the extraction of bioactive compounds depends significantly on the type of solvent, time, extraction method, and their respective interactions.

**Figure 1:**
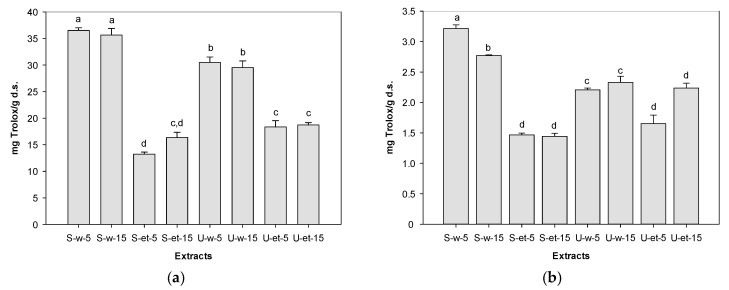
Antioxidant activity of extracts based on factorial design extraction. (**a**) ABTS; (**b**) DPPH. Different letters indicate significant differences, according to the Tukey test (*p* < 0.05). S: stirring; U: ultrasound; w: water; et: ethanol; 5 and 15: time (min).

Table 2 and Figure 1 show the antioxidant activity of the eight extracts based on the factorial design, including the variables (method, solvent, and time) and their results of antioxidant activity with ABTS and DPPH methods.

The highest antioxidant activity was observed in the aqueous extract obtained by stirring for 5 min (S-w-5), with values of 36.51 ± 0.51 mg Trolox/g d.s. and 3.21 ± 0.06 mg Trolox/g d.s., as applied to the ABTS and DPPH radicals, respectively. Followed by the aqueous extract obtained by stirring for 15 min (S-w-15) (35.64 ± 1.25 mg Trolox/g d.s. and 2.77 ± 0.01 g Trolox/g d.s., for ABTS and DPPH, respectively), the activity was very similar to the S-w-5 treatment, although, in the DPPH radical, these two treatments were significantly different (*p* < 0.05) (Figure 1b). Conversely, the lowest antioxidant capacity was shown by the S-et-5 treatment (13.23 ± 0.38 mg Trolox/g d.s.) in the ABTS radical and S-et-15 (1.44 ± 0.05 mg Trolox/g d.s.) in DPPH. Despite showing a similar tendency in the results among both radicals, the extracts had higher antioxidant capacity values with the ABTS radical than when analyzed by DPPH. These findings resemble the antioxidant capacity of *Tecoma stans* reported by Aarland et al. [43], with greater antioxidant capacity displayed by the ABTS radical than DPPH (15.02 ± 1 mg Trolox/g d.s. and 7.51 ± 0.5 mg Trolox/g d.s., respectively).

The antioxidant activity displayed by the S-w-5 treatment in the ABTS radical (36.51 ± 0.51 mg Trolox/g d.s.) was similar to that of the medicinal plants: methanolic extracts of *Buddleia officindis marim* (32.74 ± 0.65 mg Trolox/g) and *Fraxinus rhynchophylla Hance* (41.57± 0.08 mg Trolox/g) [44]; extracts in methanol, ethanol, water, and hydrochloric acid solution of *Bauhinia macrostachya* (33.36 ± 0.65 mg Trolox/g f.w.) and *Cecropia palmata* (27.25 ± 0.22 mg Trolox/g f.w.) [45,46]; and methanolic extracts of *Stachys tmolea* (32.34 ± 1.76 mg eq Trolox/g dp) [46]. Regarding the DPPH radical, the results are comparable to ethanolic extracts by stirring and performing an ultrasound of *Suaeda fruticosa* (L.) *Forssk* (5.69 mg TE/g extract) [47].

**Figure 2:**
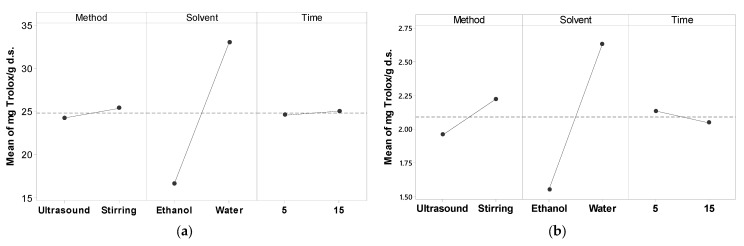
Plots of main effects for antioxidant activity (**a**) ABTS; (**b**) DPPH.

Figure 2 illustrates the effect of method, solvent, and time extraction on antioxidant activity. In both methods, the solvent was the most significant factor, and level 2 (water) had a greater effect on antioxidant capacity. Additionally, a slight significant effect of the extraction method was observed in the DPPH method (Figure 2b).

**Figure 3:**
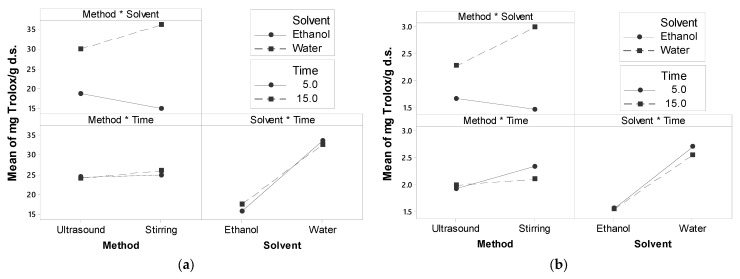
Plots of interaction effects for antioxidant activity (**a**) ABTS; (**b**) DPPH.

From the interaction plots (Figure 3), the greater the slope and the difference between points, the greater the effect or interaction [48]. The method*solvent and solvent*time indicate a strong interaction among factors in both radicals. Hence, the highest antioxidant activity was found in the extract in level 2 of the extraction method and level 2 of solvent (stirring and water, respectively) for radicals ABTS (36.51 ± 0.51 mg Trolox/g d.s.) and DPPH (3.21 ± 0.06 mg Trolox/g d.s.) (Figure 1). This treatment (S-w-5) was selected to be encapsulated by spray drying. From now on, the term *extract* will refer to this treatment extraction.

**Figure 4:**
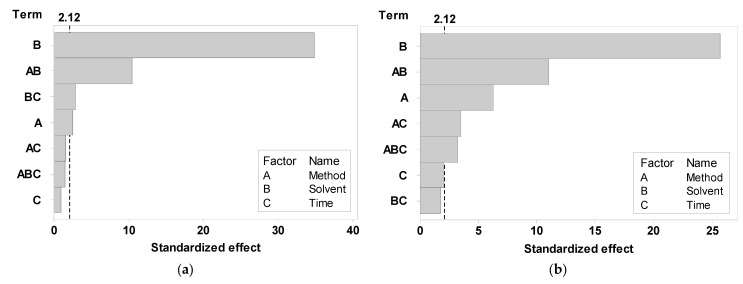
Pareto chart of the significance rank of main and interaction effects of extraction factors on antioxidant activity. (**a**) ABTS; (**b**) DPPH.

The Pareto chart indicated the following sequence of the main and interaction effects of extraction factors on antioxidant activity. For ABTS: B (solvent) > AB (method and solvent) > BC (solvent and time) > A (method) > AC (method and time) > ABC (method, solvent, and time) > C (time), while B (solvent) > AB (method and solvent) > A (method) > AC (method and time) > ABC (method, solvent, and time) > C (time) > BC (solvent and time) for the DPPH radical (Figure 4).

### 3.2. Microencapsulates Bioactive Properties

#### 3.2.1. Antioxidant Activity

**Figure 5:**
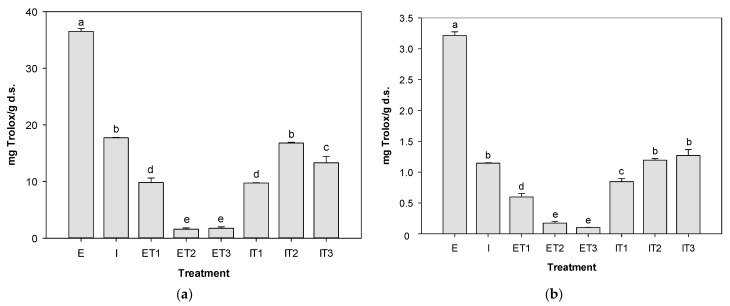
Antioxidant activity of extract, infusion, and microencapsulates. (**a**) ABTS; (**b**) DPPH. Different letters indicate significant differences, according to the Tukey test (*p* < 0.05). E: extract; I: infusion; T1: maltodextrin; T2: arabic gum; T3: maltodextrin–arabic gum.

Figure 5 shows the antioxidant activity of the extract, infusion, and the spray-dried microencapsulates of the extract and infusion of *Tecoma stans* leaves. No microencapsulate nor the infusion surpassed the antioxidant activity of the extract (S-w-5) in the ABTS and DPPH methods (36.51 ± 0.51 mg Trolox/g d.s. and 3.21 ± 0.06 mg Trolox/g d.s., respectively). Regarding the ABTS radical, the infusion (17.73 ± 0.06 mg Trolox/g d.s.) had the second highest activity, whereas for the DPPH radical, it was the infusion encapsulated with MD:AG (IT3), whose activity is statistically similar (*p* < 0.05) to the encapsulated infusion with AG (IT2) (1.19 ± 0.02 mg Trolox/g s.s.), and the infusion (1.15 ± 0.01 mg Trolox/g d.s.).

The infusion of plants is a relevant extraction method that eliminates the use of organic solvents. In addition, by obtaining nontoxic extracts with antioxidant capacity through this method, the isolation and purification of the antioxidant compounds is unnecessary [49]. The results of the antioxidant capacity of the *Tecoma stans* infusion are comparable to the reported activity in the ABTS method of the infusions from the leaves of medicinal Amazonian plants: *Anacardium excelsum* (14.67 ± 0.93 mg Trolox/g d.s.) and *Piper putumayoense* (22.78 ± 3.08 mg Trolox/g d.s.) [50]. Likewise, they align with some of the presented values of the 223 plant infusions analyzed, such as *Akebia trifoliata* (*Thunb.*) *Koidz* (17.21 ± 1.92 mg Trolox/g d.s.), *Amomum tsao-ko Crevostet Lemarie* (15.69 ± 0.18 mg Trolox/g d.s.), and *Artemisia apiacea Hance* (14.32 ± 0.57 mg Trolox/g d.s.) [51].

Regarding the assessment of the spray drying microencapsulation assessment, an evident decrease in the antioxidant activity of the extracted microencapsulates was observed compared to the extract. This might be due to the high injection temperature in the drying process causing the degradation of compounds like phenolics and subsequently the antioxidant capacity [52], as well as the amount of the *T. stans* sample used in the extract. Nonetheless, encapsulation with MD was able to preserve considerably better the extract antioxidants in both radicals (9.89 ± 0.82 mg Trolox/g d.s. and 0.6 ± 0.06 mg Trolox/g d.s. for ABTS and DPPH, respectively). This agrees with the results reported by Sarabandi et al. [53], in which an encapsulated eggplant peel extract produced with MD had higher antioxidant capacity than that with AG, while Abdel-Aty et al. [54] found that the use of gum and maltodextrin as microencapsulation coats favored the retention of antioxidant compounds such as the phenolic content of garden cress. Furthermore, the selection of coating material has a considerable impact on the properties of the encapsulates including solubility, stability, and quality.

Contrariwise, the infusion microencapsulates produced with AG (IT2) (16.8 0.13 mg Trolox/g d.s.) in ABTS and AG (IT2) (1.19 0.02 mg Trolox/g d.s.) and MD:AG (IT3) (1.27 0.1 mg Trolox/g d.s.) in DPPH preserved considerably the antioxidants in the *T. stans* infusion (Figure 5), without significant differences between treatments being observed (*p* < 0.05). Maltodextrin is widely used in bioactive compound encapsulation because of its characteristics such as high solubility, low viscosity at high solid concentrations, low cost, and core protection against oxidation. However, since it has a low emulsifying ability, it is mixed with other agents to form more stable emulsions [55], as evidenced in the results.

#### 3.2.2. Hypoglycemic Activity

The released glucose in the extract, infusion, microencapsulates, and white bread was determined with the glucose oxidase enzyme. This enzyme is widely used to assess blood glucose levels due to its high specificity with glucose [56].

The blood glucose response is represented by the area under the curve of every sample shown in Table 3. The highest hypoglycemic activity was noticed in the microencapsulated infusion with MD:AG (IT3) (8218.40 ± 28.8 mg/dL), followed by the IT1 (8825.20 ± 53.2 mg/dL), IT2 (9001.90 ± 62.3 mg/dL), infusion (9949.80 ± 32.3 mg/dL), ET3 (10,676.50 ± 51.9 mg/dL), ET1 (11,001.20 ± 40.9 mg/dL), and ET2 (11,081.20 ± 18.5 mg/dL) treatments. The spray drying encapsulation caused a considerable decrease in the glucose released levels in comparison to the non-spray-dried samples. This improvement in hypoglycemic activity can be attributed to the encapsulation process, as it is acknowledged as a method to preserve bioactive compounds from undesirable surrounding conditions and improve their beneficial effects, availability, and efficacy [57]. MD:AG as wall material presented the best results in such improvement: from 12,407.40 ± 39.5 mg/dL to 10,676.50 ± 51.9 mg/dL (ET3) for the extract and from 9949.80 ± 32.3 mg/dL to 8218.40 ± 28.8 mg/dL (IT3) for the infusion. This same tendency was found in Khalifa et al.’s study, which reported that the encapsulated blackberry juice with MD and MD:AG showed the most significant retention of α-glucosidase activity [58]. The combination of agents like MD and AG is a common strategy to improve encapsulation by providing better emulsification properties that result in more stable encapsulation systems [57].

The powder’s stability in this study was related to the carrier combination (MDX, AG). This generates the formation of structures denser and more continuous in the capsules preventing oxygen transfer through the system and thus retard the oxidation of bioactive compounds [59].

The lowest glycemic index was observed in the microencapsulated infusion with MD:AG (IT3) (GI = 47) (Table 3). According to the ISO standard, this powder would be categorized as a low glycemic index product [36]. Furthermore, this GI value is comparable to medicinal plant extracts with antidiabetic effects such as *Suropus androgynus* (GI = 55) and *Tinospora cordifolia* (GI = 39) [60].

### 3.3. Microencapsulates Characterization

Only the powders that were shown to preserve the bioactive properties of *T. stans* were selected for the characterization phase. These microencapsulates were the extract encapsulated with MD (ET1) and the infusion encapsulated with MD:AG (IT3). The former preserved the antioxidant activity of the extract, while the latter preserved the hypoglycemic activity of the infusion.

#### 3.3.1. Physicochemical Characterization

Table 4 presents the results of the physicochemical analysis. The yield percentages of the ET1 and IT3 powders were statistically similar (*p* < 0.05): 18.34 ± 3.13 and 21 ± 10.47, respectively. These percentages are close to the results reported by Jiménez-González et al. [59] of the *Renealmia alpinia* pigments encapsulation with MD (17.17 ± 0.2) and MD:AG (18.01 ± 4.2).

The moisture content in a microcapsule determines the stability during storage and is correlated to aw, flowability, stickiness, drying efficiency, bioactive compound oxidation, and microbial growth [61]. Meanwhile, no significant differences were observed in the moisture percentage of the ETI (4.04 ± 0.2%) and IT3 (4.75 ± 0.63%) microencapsulates; the water activity was statistically different (*p* < 0.05). The produced powders were microbiologically stable as aw values in a 0.2–0.4 range assure product stability [62].

The wettability time was statistically higher (*p* < 0.05) in IT3 (460 ± 2 s) than in ET1 (297.33 ± 14.29 s). This might be explained by the larger MD concentration in ET1. Maltodextrin acts as a volume agent that affects porosity, and as porosity increases, wettability does as well [63]. This effect was described by Caliskan et al.’s [62] research, which noticed that an increment in maltodextrin concentration in the sumac spray drying caused a significant decrease in wettability times.

Regarding the densities, no significant differences were found (*p* < 0.05) in both microencapsulates. Bulk density depends on particle properties such as size, shape, and surface. Tapped density influences the package, transport, and commercialization of powders [61,64].

The powders of white color exhibited high L* values ranging from 88 to 90 at first glance. The luminosity (L*) was significantly lower (*p* < 0.05) in the microencapsulated extract with only MD. Previous studies have also reported decreased L* with MD as the wall material [65]. Likewise, significant differences (*p* < 0.05) were observed among powders in a* and b* parameters.

#### 3.3.2. Compound Identification

A total of 57 different compounds in the extract microencapsulates and 25 in the infusion microencapsulates were found using gas chromatography/mass spectrometry. Table 5 shows the identified compounds with area percentages higher than 5%. The compounds found in higher concentrations for the microencapsulated extract were as follows: gibberellic acid and 2-furanmethanol in ethanol and 1-hexene,3,4-dimethyl and butane, 2,2-dimethyl in hexane. Whereas, for the microencapsulated infusion: gibberellic acid and 2-furanmethanol in ethanol; butane, 2,2-dimethyl in hexane; and benzene, 1,3-bis (3-phenoxyphenoxy) and (2,3-diphenyl-cyclopropyl) methyl phenyl sulfoxide, trans, in ethyl acetate.

Gibberellic acid, one of the main compounds found in chromatograms, is a phytohormone that regulates relevant aspects of plants such as growth, germination, elongation, and flowering. In humans, this compound comes from the intake of fruits and vegetables, and there is little information on its effect [66,67].

2-furanmethanol has been identified in the honey Greek-type Fir, to which antiestrogenic effects are attributed, furthermore exhibiting antioxidant activity [68,69].

Similarly, nerol is reported to possess antioxidant, antibacterial, and antifungal activity. Even though recent research has rarely reported its anti-diabetic effect, this monoterpenoid has been identified in cinnamon (Cinnamon verum), to which anti-diabetic and other beneficial properties have been demonstrated [70,71,72].

### 3.4. Micrographs of Microencapsulates

**Figure 6:**
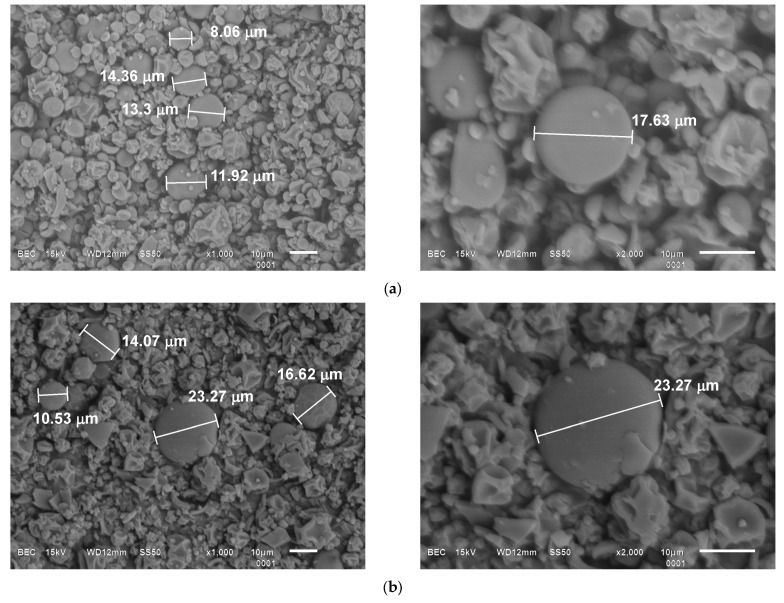
Size and morphology of microcapsules. (**a**) Microcapsules of spray-dried extract with MD (ET1); (**b**) microcapsules of spray-dried infusion with MD:AG (IT3).

Figure 6 shows the micrographs of powders. The average size of the microcapsules was 13.05 ± 3.13 μm and 16.12 ± 4.66 μm, for ET1 and IT3, respectively, with no significant differences in samples (*p* < 0.05). Regarding the morphology, both powders presented spherical capsules and, in a more considerable proportion, irregular, collapsed capsules with dents on their surface. At first glance, the microcapsules with a regular appearance of ET1 (Figure 6a) displayed a more defined shape. This may result from the more significant concentration of MD in ET1 since it generally confers better protection against thermal degradation by enhancing the glass transition temperature (Tg) of wall materials, pre-venting the collapse of capsules [55].

## 4. Conclusions

The present study allowed us to determine that solvent and extraction methods were the factors that most influenced the antioxidants extraction of *T. stans* leaves based on the factorial design analysis. Slight differences, yet the same tendency of the antioxidant activity (AA) results with the ABTS and DPPH methods, were observed. The best extraction conditions were water, stirring for 5 min.

Following the spray drying, the AA of the extract decreased significantly, likely because of high injection temperatures in the spray drying process. Contrarily, the AA of the infusion was preserved after the encapsulation process. Regarding the hypoglycemic activity, the spray-dried samples exhibited higher activity after the microencapsulation, where the combination of agents (MD:AG) performed better in the extract and infusion. In addition, the antidiabetic activity of the *T. stans* infusion taken in traditional medicine was demonstrated.

The characterization of analyzed powders indicated significant differences in water activity, wettability, and color. Additionally, gibberellic acid, 2-furanmethanol, and nerol were some of the main identified compounds via gas chromatography/mass spectrometry.

Thus, the microencapsulates of *Tecoma stans* obtained via spray drying in this research have the potential to be further investigated to eventually be used as functional ingredients that enhance the nutritional properties of food matrices, in turn helping in the prevention/treatment of chronic diseases like diabetes.

## Figures and Tables

**Table 1:** Factors and levels used in the factorial design.

Factor	Level 1 (−1)	Level 2 (1)
Method	Ultrasound	Stirring
Solvent	Ethanol	Water
Time (min)	5	15

**Table 2:** Antioxidant activity according to the factorial analysis, considering the variables method, solvent, and time.

			Antioxidant Activity (mg Trolox/g d.s.)	
Method	Solvent	Time	ABTS	DPPH
−1	−1	−1	18.34 ± 1.19	1.65 ± 0.14
1	−1	−1	13.23 ± 0.38	1.47 ± 0.03
−1	1	−1	30.49 ± 1.02	2.21 ± 0.03
1	1	−1	36.51 ± 0.51	3.21 ± 0.06
−1	−1	1	18.72 ± 0.44	2.24 ± 0.08
1	−1	1	16.36 ± 0.99	1.44 ± 0.05
−1	1	1	29.55 ± 1.22	2.33 ± 0.10
1	1	1	35.64 ± 1.25	2.77 ± 0.01

**Table 3:** Areas under the curve of the released glucose and glycemic index (GI).

	Area under the Curve		GI
	mg/dL	mmol/L	
White bread	12,379.80 ± 33.8 a	688.38 ± 1.9 a	71 a
Extract	12,407.40 ± 39.5 a	689.95 ± 2.3 a	71 a
Infusion	9949.80 ± 32.3 d	553.48 ± 1.4 d	57 e
ET1	11,001.20 ± 40.9 b	613.67 ± 4.0 b	63 b
ET2	11,081.20 ± 18.5 b	616.28 ± 1.0 b	64 d
ET3	10,676.50 ± 51.9 c	593.37 ± 2.3 c	61 c
IT1	8825.20 ± 53.2 f	490.78 ± 3.0 f	51 f
IT2	9001.90 ± 62.3 e	501.33 ± 2.9 e	52 f
IT3	8218.40 ± 28.8 g	456.95 ± 1.6 g	47 g

Different letters in the same column indicate significant differences, according to the Tukey test (*p* < 0.05). E: extract; I: infusion; T1: maltodextrin; T2: arabic gum; T3: maltodextrin–arabic gum.

**Table 4:** Physicochemical properties.

	ET1	IT3
Yield (%)	18.34 ± 3.13 a	21.98 ± 10.47 a
Moisture (%)	4.04 ± 0.4 a	4.75 ± 0.63 a
Aw	0.298 ± 0.001 a	0.231 ± 0.003 b
Wettability (s)	297.33 ± 14.29 b	460 ± 2 a
Bulk density (g/mL)	0.261 ± 0.003 a	0.257 ± 0.006 a
Tapped density (g/mL)	0.375 ± 0.008 a	0.371 ± 0.013 a
Color		
L*	88.627 ± 0.006 b	90.61 ± 0.017 a
a*	0.453 ± 0.025 a	−8.3 ± 0.01 b
b*	9.007 ± 0.015 b	18.427 ± 0.025 a

Different letters in the same row indicate significant differences, according to the Tukey test (*p* < 0.05). ET1: encapsulated extract with MD; IT3: encapsulated infusion with MD:AG.

**Table 5:** Characterization of *Tecoma stans* microencapsulates identified by GC-MS.

Microencapsulate	Solvent	Chemical Compounds	Area (%)
Extract T1	Ethanol	Gibberellic acid	30.32
		Cyclobutane, 1,1-dimethyl-2-octyl	11.89
		2-Furanmethanol	27.68
	Hexane	Butane, 2,2-dimethyl	9.58
		1-Hexene,3,4-dimethyl	13.56
		Cyclopentane, methyl	6.73
Infusion T3	Ethanol	Gibberellic acid	29.86
		2-Furanmethanol	14.25
	Hexane	Butane, 2,2-dimethyl	23.63
	Ethyl acetate	Benzene, 1,3-bis (3-phenoxyphenoxy)	13.37
		Nerol	5.05
		Propanoic acid, 2-(3-acetoxy-4,4-14-trimethylandrost-8-en-17-yl-	7.37
		(2,3-diphenyl-cyclopropyl) methyl phenyl sulfoxide, trans)	12.59

T1: maltodextrin; T3: maltodextrin–arabic gum.

## Data Availability

The original contributions presented in the study are included in the article, further inquiries can be directed to the corresponding authors.
